# Erectile Dysfunction and Associated Anxiety and Depression in Male Hemodialysis Patients: A Cross-Sectional Study at Karachi Institute of Kidney Diseases

**DOI:** 10.7759/cureus.11129

**Published:** 2020-10-24

**Authors:** Furqan Ahmad Jarullah, Mahjabeen Yaseen, Hassan Abdullah, Sadia Yaqoob

**Affiliations:** 1 Medicine, Jinnah Medical and Dental College, Karachi, PAK; 2 Nephrology, Fazaia Ruth Pfau Medical College, Karachi, PAK; 3 Neurology, University of Alabama, Birmingham, USA; 4 Medicine, Nishtar Medical University, Multan, PAK

**Keywords:** hemodialysis, erectile dysfunction, depression, anxiety

## Abstract

Objectives

Chronic kidney disease (CKD) is one of the most prevailing diseases in the world and is associated with sequelae of depression, anxiety, and sexual dysfunction. The goal of our study is to measure the prevalence of erectile dysfunction, depression, and anxiety among patients suffering from CKD and to establish a correlation between them.

Methodology

The research was a single-centered, descriptive cross-sectional study. All male patients present at the time of the survey were interviewed, and then based on the inclusion and exclusion criteria, 84 were selected. The questionnaire comprised demographic variables, erectile function scoring using the International Index of Erectile Function (IIEF) scale, and Hospital Anxiety and Depression Scale (HADS) for depression and anxiety. All data were analyzed using SPSS Software 25.0 (IBM Corp., Armonk, USA).

Results

Out of 84 male patients, 47.6% had erectile dysfunction (ED). 10% of the affected individuals had depression and 3% reported having anxiety. No known external factors had any contribution to erectile malfunction, depression, and/or anxiety.

Conclusion

We found no correlation of depression and/or anxiety with ED in this population of male subjects undergoing hemodialysis.

## Introduction

Chronic kidney disease (CKD) is an emerging crisis in the current time. According to a study published in December 2016 by the National Institute of Diabetes and Digestive and Kidney Diseases in the US, there is a 14% prevalence of CKD in the US citizens [1]. Due to very few studies conducted in this area in Pakistan, we have insufficient data to determine the actual prevalence of the disease. Chronic renal failure (CRF) is now a public health problem, mainly because of its prevalence, evolution, and the fact that it is the complete antonym of cost-effective and causes a financial burden on patients and the healthcare system [2]. The only two possible methods of treatment are lifelong hemodialysis or renal transplantation. As the majority of the population cannot afford renal transplants and the fact that very few donors are available, patients have no choice but to undergo hemodialysis as much as up to three times per week for 3-4 hours for the rest of their lives [3].

There have been reports of multiple obstacles that these patients have to face in their daily lives, apart from the physical and mental torture brought upon them by society. A common complaint that affects their quality of life is erectile dysfunction (ED). In 2014, approximately 150 million males were affected by ED worldwide and projected for numbers to double by 2025 [4]. It is defined as the persistent inability to achieve and/or maintain an erection enough for satisfactory sexual intercourse [5]. Scientists first documented in 1975 that erectile dysfunction was closely associated with hemodialysis [3]. It is said that a combination of organic and psychological factors is attributable to this condition in a human being [6]. Such factors required for normal erections include a complex balance of physiological, hormonal, neurological, and vascular factors, as well as emotional and social factors [7]. Sexual satisfaction, on the other hand, also seems to be a problem for these patients. Sexual gratification is defined as physical or emotional satisfaction provided by self-regulation [8].

ED is not only a medical problem but affects the marital relations and patient's self-esteem and causes the quality of life to decrease. which in turn causes the patient to go into depression or develop anxiety. Considering all these circumstances and horrors the patients go through, many of them spiral their way into depression and anxiety. However, very few studies have been done to correlate ED with depression and anxiety. Since these conditions have such a vital impact on daily activities, it tends to falter the quality of life of these patients, indirectly declining it. It can be said confidently that in older patients, presentation for dialysis has a later onset, with numerous co-morbidities. It is believed that there is an increased risk of cognitive dysfunction, increased vulnerability to infections, and sensory impairments as well as functional and psychological dependence in these patients [9].

Objectively, this study was conducted to create, or rather, build the foundation to establish the link between sexual dysfunction in male patients who undergo hemodialysis and whether it has a connection with these patients undergoing anxiety and/or depression. Studies conducted so far have not been able to fill this gap in knowledge, especially in our developing country. Furthermore, Procci et al. observed that sexual dysfunction is not associated with primary kidney disease [10]. Hence, one other purpose to conduct this research was to follow up on his vision since a lot has changed since 1981 [10].

## Materials and methods

Study setting

This study was conducted in the setting of the Karachi Institute of Kidney Diseases located in the Federal B Area of Karachi, Pakistan.

Subjects, sample size, and data analysis

The study design selected was cross-sectional and it was conducted in November 2019. Our study includes adults who were suffering from chronic kidney disease and were undergoing hemodialysis in this institute. All male patients within that institute undergoing renal replacement therapy were surveyed. A total of 107 males were admitted at that time. Only 84 participants were included in this study as per the inclusion and exclusion criteria mentioned later. We used SPSS version 25.0 to analyze our data.

Data collection tools

A questionnaire was developed which contained questions regarding socio-demographic data, depression, and anxiety scoring according to Hospital Anxiety and Depression Scale (HADS), and sexual dysfunction scoring according to the International Index of Erectile Function (IIEF) [11].

Relevant socio-demographic data included age, number of children, occupation, duration of CRF and hemodialysis, number of dialysis sessions per week, and recent blood work including levels of hemoglobin, calcium, and phosphorous. HADS scale includes seven questions each for assessment of anxiety and depression. IIEF scale assesses sexual function by a series of 15 questions divided into five domains, namely erectile function, orgasmic function, sexual desire, intercourse satisfaction, and overall satisfaction.

Inclusion and exclusion criteria

Married males of 18 years or older were included in this study. Any participant who was under the legal age of marriage, which is 18 years (in Pakistan), unmarried, has not been living with their spouse for at least last one month, widower, suffering from severe co-morbidity such as paralysis or psychiatric disease, and demised during the study were excluded.

Ethical considerations

Each interview began with careful consideration of privacy and verbal informed consent that the questions asked will not be disclosed to anyone. A letter of approval was taken from the ethics committee of the institute.

## Results

Among the 84 men, concerning the International Index of Erectile Function (IIEF), erectile dysfunction (ED) prevailed in 40 (47.6%). The average age of the total sample was 48.7 ± 12.5 years, whereas those with ED had an average age of 51.1 ± 12.5 years, the youngest being at an age of 27 years and the oldest being 74 years of age. 27 (67.5%) of the affected individuals seemed to have severe ED. The mean of the total IIEF score was 47.4 ± 20.0.

Table 1 shows the demographics and clinical parameters of patients enrolled in the study.

**Table 1:** Demographics and clinical parameters of patients NSAIDS: non-steroidal anti-inflammatory drugs; Hb: hemoglobin

Variables	
Patients demographics	
Age (years) mean ± SD	48.7 ± 12.5
BMI (kg/m^2^) mean ± SD	23.9 ± 4.5
Duration of hemodialysis (years) mean ± SD	2.32 ± 2.4
Cause of renal failure ( %)	
Diabetes mellitus	35.7
Hypertension	57.1
Glomerulonephritis	6
Renal stone disease	11.9
NSAIDs	11.9
Cystic kidney disease	3.6
Other diseases (%)	
Hepatitis B	4.7
Hepatitis C	17.8
Cardiovascular diseases	2.3
Patient clinical characteristics	
Hb (g/l)	10.9 ± 2.1
Calcium (mmol/l)	8.25 ± 1.3
Phosphorous (mmol/l)	5.14 ± 1.8
Albumin (g/l)	3.9 ± 0.89

Their mean age was 48.7 ± 12.5 years, while the average duration of hemodialysis was 2.32 ± 2.4 years. It was also seen that 28 (70%) of the affected individuals were anemic, whereas 33 (75%) of the individuals who did not have ED were anemic. Pearson’s correlation was done for this particular variable, and that too was seen to be inconsequential (Table 2).

**Table 2:** Correlational analysis of anemia with erectile dysfunction Statistical significance (p<0.05)

Correlations			
		Anemia	Erectile dysfunction
Anemia	Pearson Correlation	1	.056
	Sig. (2-tailed)		.613
	N	84	84
Erectile dysfunction	Pearson Correlation	.056	1
	Sig. (2-tailed)	.613	
	N	84	84

**Figure 1:**
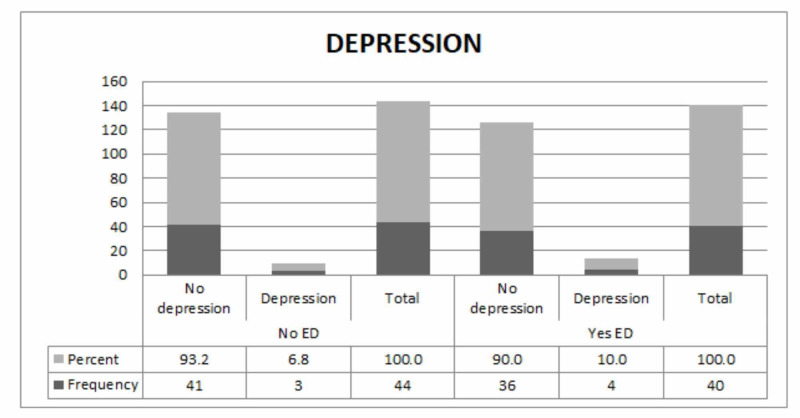
Frequency of depression in the study participants ED: erectile dysfunction

**Figure 2:**
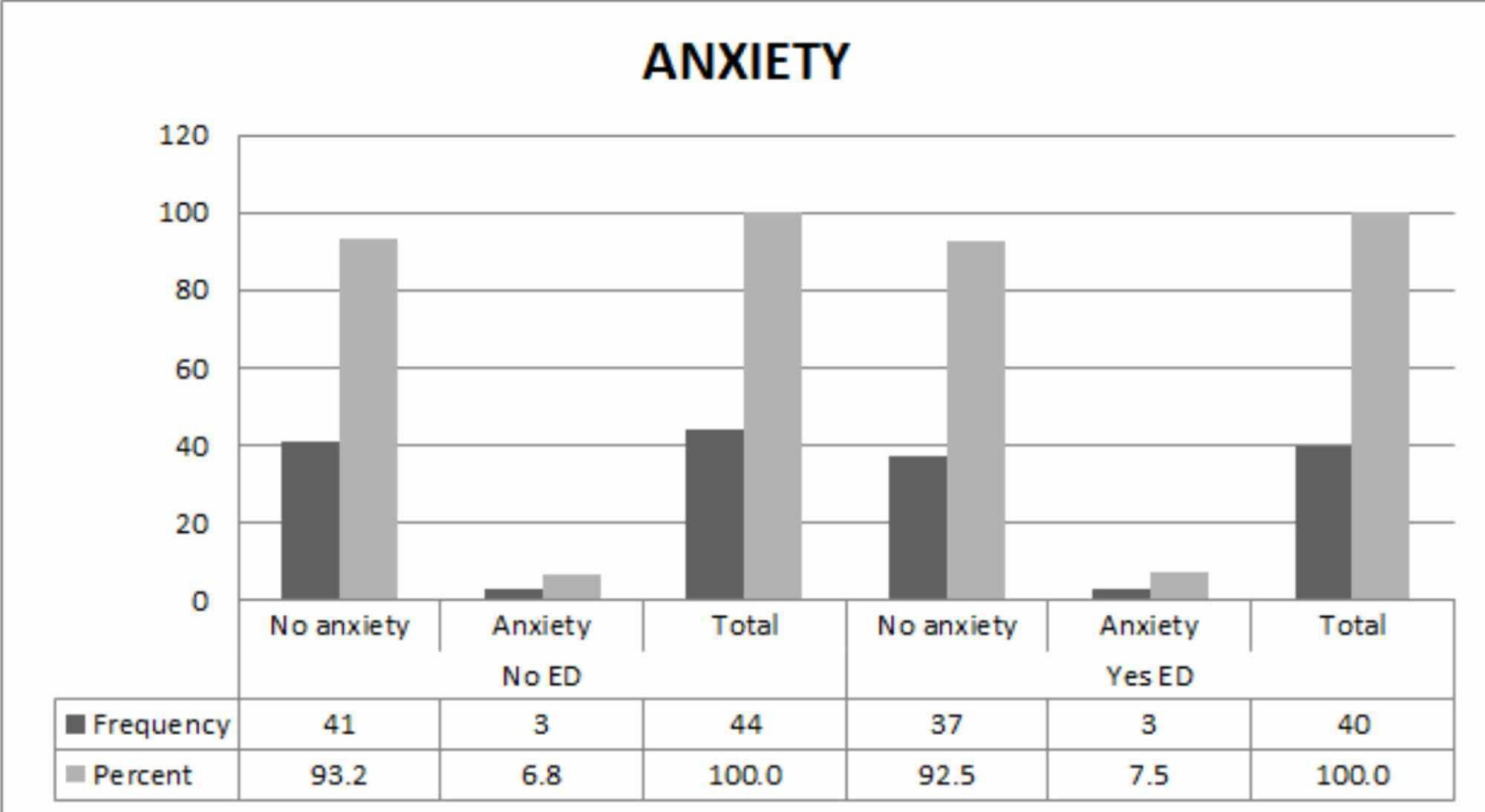
Frequency of anxiety in the study participants ED: erectile dysfunction

A total of 16.8% of the participants had depression, of which only 10% were suffering from ED. Anxiety, however, prevailed in 14.3% of the study population and about 7.5% had erectile dysfunction. Figure 1 and Figure 2 show the frequency of depression and anxiety in the study participants, respectively.

Pearson’s correlation suggests a positive correlation, but p-values were negligible with the values being 0.905 and 0.603 for anxiety (Table 3) and depression (Table 4), respectively.

**Table 3:** Correlational analysis between anxiety and erectile dysfunction These correlations show positivity but without any significant p-values. Significant p-value (p<0.05)

Correlations			
		Anxiety	Erectile dysfunction
Anxiety	Pearson Correlation	1	.013
	Sig. (2-tailed)		.905
	N	84	84
Erectile dysfunction	Pearson Correlation	.013	1
	Sig. (2-tailed)	.905	
	N	84	84

**Table 4:** Correlational analysis between depression and erectile dysfunction These correlations show positivity but without any significant p-values. Significant p-value (p<0.05)

Correlations			
		Erectile dysfunction	Depression
Erectile dysfunction	Pearson Correlation	1	.057
	Sig. (2-tailed)		.603
	N	84	84
Depression	Pearson Correlation	.057	1
	Sig. (2-tailed)	.603	
	N	84	84

Hence, we failed to reject the null hypothesis that there is no association between erectile dysfunction and associated anxiety and depression in male hemodialysis patients.

## Discussion

Yu-Sen Peng et al. quoted the mean total IIEF score to be 49.1 ± 17.6, whereas our research shows a slightly lower mean total of 47.4 ± 20.0. The difference is probably suggestive of the variation in sample size being 154, almost double as compared to ours [12].

It was noted in this study that the prevalence of ED in males was lower (47.6%) as compared to the study done by Antonucci et al. who reported 70% ED [3]. Gorsane et al. reported 80% ED and Arslan et al. recorded an 80.7% prevalence of ED in males undergoing hemodialysis [13,6]. However, it was intriguing to discover that studies conducted way back in the 1970s like Abram et al. and those in the 1980s like Rodger et al. also noted the same pattern where ED prevailed in more than 50% of the patients [14,15].

Possibly, the prevalence could be even much less had it not been for about a handful of patients who were not indulging in any sort of sexual activity to prevent the spread of other co-morbidities like hepatitis C to their spouses. There were a total of 15 (17.9%) candidates who had hepatitis C and only 5 (33.3%) of these suffered from ED. A total of four participants also had hepatitis B and three (75%) of them were co-morbid with ED. It is stated that ED may have an increased incidence with the duration of hemodialysis; however, there has been no significant finding in this research that could support this theory.

Baldwin et al. study shows that there is an association of patients diagnosed with depression to have erectile dysfunction; however, this is not supported in this study [16]. Guven et al. did find a correlation between anxiety and education level, however, we could not tabulate any such findings with any of the known parameters [17]. Although there does seem to be some sort of positive correlation, there is no apparent significance to the theory, as shown in the research conducted by Esen et al. [18]. Paraskevi et al. did a study that revealed a negative correlation between ED and depression [19].

Theofilou et al. not only proved negative correlations between ED and depression but also anxiety proved to be significantly negatively correlated [19].

## Conclusions

This study gives rise two main conclusions, namely, prevalence rate and association with depression and anxiety. We conclude that the prevalence rate is much lower than reported in other countries and depression and/or anxiety are not significantly correlated with erectile dysfunction. ED is seemingly thriving, and to find a light for this dwindling staircase into depression, anxiety, and maybe even infertility by natural methods is fundamental.

This study was conducted in a single center and comprised a fairly small number of patients. Nonetheless, the findings prompt multiple studies to verify and confirm that ED in the Pakistani population is relatively lower in hemodialysis patients as well as that it is not significantly linked to depression and anxiety. Follow-up studies are needed in the future for clearer results. 
